# Application of Encapsulation Strategies for Probiotics: From Individual Loading to Co-Encapsulation

**DOI:** 10.3390/microorganisms11122896

**Published:** 2023-11-30

**Authors:** Sofia Agriopoulou, Maria Tarapoulouzi, Theodoros Varzakas, Seid Mahdi Jafari

**Affiliations:** 1 Department of Food Science and Technology, University of the Peloponnese, 24100 Kalamata, Greece; s.agriopoulou@uop.gr; 2 Department of Chemistry, Faculty of Pure and Applied Science, University of Cyprus, P.O. Box 20537, Nicosia CY-1678, Cyprus; tarapoulouzi.maria@ucy.ac.cy; 3 Department of Food Materials and Process Design Engineering, Gorgan University of Agricultural Sciences and Natural Resources, Gorgan 49189-43464, Iran; smjafari@gau.ac.ir; 4 Halal Research Center of IRI, Iran Food and Drug Administration, Ministry of Health and Medical Education, Tehran 14158-45371, Iran

**Keywords:** encapsulation, bioactive ingredients, probiotics, nanoemulsions, co-encapsulation

## Abstract

Consumers are increasingly showing a preference for foods whose nutritional and therapeutic value has been enhanced. Probiotics are live microorganisms, and their existence is associated with a number of positive effects in humans, as there are many and well-documented studies related to gut microbiota balance, the regulation of the immune system, and the maintenance of the intestinal mucosal barrier. Hence, probiotics are widely preferred by consumers, causing an increase in the corresponding food sector. As a consequence of this preference, food industries and those involved in food production are strongly interested in the occurrence of probiotics in food, as they have proven beneficial effects on human health when they exist in appropriate quantities. Encapsulation technology is a promising technique that aims to preserve probiotics by integrating them with other materials in order to ensure and improve their effectiveness. Encapsulated probiotics also show increased stability and survival in various stages related to their processing, storage, and gastrointestinal transit. This review focuses on the applications of encapsulation technology in probiotics in sustainable food production, including controlled release mechanisms and encapsulation techniques.

## 1. Introduction

Encapsulation is defined as an entrapment process of one substance, bacteria, or bioactive compounds (bioactives) inside another one, thus producing particles with a diameter of a few nm to a few μm. The substance that is encapsulated may be called the core material, active agent, or internal phase. A coating, membrane, shell, carrier material, wall material, external phase, or matrix could define the encapsulating substance [1,2,3]. Another definition of encapsulation states “the preparation of solid, liquid or even gaseous components in carriers-microcapsules and the active release of their contents in a controlled rate under the influence of defined conditions” [4]. Capsules can be classified into three types based on their sizes: nano- (<0.2 μm), micro- (0.2–5000 μm), and macro- (>5000 μm) capsules. Micro- and nanoencapsulation are the most common types of encapsulation in food technology [4]. The shape of these capsules is usually spherical or ellipsoidal, and many factors affect it such as the structural characteristics of the carrier, the encapsulated bioactives, the preparation method, and the drying conditions.

Encapsulation has wide applications in food, pharmaceutical, and cosmetic industries but also in metrology science, analytical chemistry, and nanoelectronics. The ability to incorporate new functional features into industrial foods and to deliver effective action is the main advantage of this technology [4]. Protecting the bioactives from various environmental factors, e.g., light, heat, oxygen, moisture, and interaction with the packaging material which could oxidize and degrade the ingredient in question, is one of the major aims of encapsulation. The instability of bioactives may also be due to industrial process conditions [5]. Masking undesirable odors produced by vitamins, minerals, and other bioactives, masking unpleasant tastes, increasing product stability, and extending its shelf life are amongst other equally important objectives of encapsulation [6]. The encapsulation technique also interacts with the design and development of new products, providing the possibility to impart special characteristics to food products (e.g., aroma, taste, texture, and color) [4]. 

Metchnikoff was the first to talk about probiotic bacteria and how the consumption of live microorganisms is positively related to human health, provided that it is taken in sufficient quantities [7]. Probiotics are living microorganisms found in many foods and via human consumption, beneficial properties for human health are derived [8]. The encapsulation of probiotics, in addition to its applications in food, also finds applications in ruminant diets [9]. Among the most important problems facing the efficacy of probiotics are viability and transport within the human gastrointestinal tract (GIT). Various factors may significantly reduce the beneficial effects of probiotics on the consumer. These factors are related to heat, pH, water activity (w_a_), and oxygen [10]. The encapsulation of probiotics is an important means of dealing with these problems, since it significantly improves their effectiveness, stability, and their bioavailability [11] as it creates an optimal microenvironment [12].

Encapsulating probiotic bacteria in microspheres or microcapsules helps protect them from harsh conditions during fermentation as well. Wall materials could be alginate, chitosan, gelatin or plant mucilages, whey proteins, gums [13], and polysaccharides [14], which must be inexpensive, robust, food-grade, and without antimicrobial activity [8,11,15]. Nanoparticles, such as chitosan nanoparticles, can protect probiotics and enhance their stability. These nanoparticles can also offer controlled release of probiotics during fermentation [16]. Emulsions can also be used to deliver probiotics in a stable and bioavailable form. This is particularly useful in ensuring the viability of probiotics during fermentation and in the digestive system [17].

Moreover, co-encapsulation techniques of probiotics have been mentioned in this review referring to the main relevant techniques such as spray drying, freeze drying, extrusion, coacervation, electrospraying, and emulsification [18,19]. 

The encapsulation of the probiotic *Lactobacillus plantarum* using porous starch (PS) via mild gelatinization (37 °C) has been reported by Zhu et al. [20]. Another example of individual encapsulation is that of lactoferrin addition, a milk protein used as a natural carrier, and if it is coupled to polyphenols, it can create an efficient delivery system. For example, Li et al. [21] investigated the covalent binding of protocatechuic acid (PA) and gallic acid (GA) to lactoferrin and found that these conjugates might have a high potential in the food industry. In addition, targeted delivery systems for probiotics could be stimulus-responsive smart hydrogels prepared by polysaccharides, thus helping to control the release rate and improve intestinal stability during their digestion and metabolism [22,23]. Researchers have encapsulated probiotics within biomaterials and enhanced the activity and stability of probiotics. Hence, they enabled site-specific release, overcoming technical obstacles encountered during the processing and application of probiotics. Sun et al. [12] reviewed the recent advancements in probiotics encapsulated within biomaterials and examined the materials, methods, and effects of encapsulation.

This review focuses on the applications of encapsulation technology for probiotics in sustainable food production and analyzes the encapsulation techniques and controlled release mechanisms, along with co-encapsulation methodologies.

## 2. Encapsulation Techniques

Probiotic encapsulation techniques can be categorized into two types: chemical (coacervation, ionic gelation, and molecular inclusion), and physical (spray drying, freeze drying, spray chilling, spray cooling, extrusion, fluidized bed drying, electrospraying, and electrospinning) [24]. Probiotic cells are between 1 and 5 μm in size and their viability must be maintained during the encapsulation process [25]. The main encapsulation processes for probiotics are freeze drying, spray drying, ionic gelation, complex coacervation, electrospraying, and electrospinning.

### 2.1. Spray Drying

Spray drying is mainly used to protect the heat-sensitive prebiotics and comprises a cheap and simple dehydration process being used on an industrial scale [2]. It is also used as an encapsulation technique. Bioactives are encapsulated following dissolution, emulsification, or dispersion with an aqueous or organic solution containing the encapsulating agent, homogenization with or without the use of an emulsifier, and then suspension or spraying the mixture in a drying chamber. Dry powder spirals to the bottom of the container where it is collected following evaporation of the water in the chamber [6,26,27]. The process is described as follows: I first phase includes injection via a pump of the liquid formulation (i.e., the mixture containing the solution with the wall material and bioactives) along with spraying at the entrance of the drying chamber. There, a nozzle or vaporizer that emits a stream of hot air helps to convert it into tiny droplets [6,26].

Commonly used nozzles are divided into three types: centrifugal disc sprayer, pneumatic nozzles, and pressure nozzles. The first is easy to use, whereas pneumatic nozzles are less efficient and increase costs, and pressure nozzles are suitable for drying high-viscosity solutions [20]. This is why homogenization is carried out at the initial stage, since it creates a stable solution with a low viscosity, producing smaller droplets without air being included in the particle. A high-viscosity solution may cause the formation of elongated and large droplets. This negatively affects the drying rate and therefore the spraying process [6].

Evaporation of the liquid occurs instantaneously since the size of the droplets that have been created is extremely small, resulting in an increase in their contact surface with the hot air stream. Hence, the drying process is completed at a rapid rate [6,26]. It should be emphasized that the gas with which the droplets will come into contact takes the form of either heated atmospheric air or inert gas in the case of flammable or oxygen-sensitive drying products. Finally, the particles are collected at the bottom of the conical drying chamber where they are separated from the gas stream via filtration, while the gas is expelled outside the chamber [26].

Spray drying has proven to be an effective process for the relatively large particle size of probiotics [26,28]. Proteins, starches, polysaccharides, and sugars are usually used as wall materials. The membrane is protected by trehalose, maltodextrin, gum arabic, and whey protein, hence maintaining its integrity due to the formation of bonds with membrane proteins. The result of these properties is the control of moisture content and particle size [24]. During spray drying and according to Buchi apparatus, seven principles are adopted.

1. Heating of the inlet air to the desired temperature (<220 °C).
2. Two fluid nozzles operate the droplet formation, whereas an ultrasonic spray head operates the nano spray dryer.
3. Drying chamber (heat exchange between drying gas and droplets).
4. Particle collection using cyclones. Electrostatic particle collector for the nano spray dryer.
5. Outlet filter for the collection of the finest particles.
6. Drying gas is delivered by an aspirator. An aspirator or compressed air could be used by the nano spray dryer.
7. Filtering of the drying gas.

During spray drying, a thermally induced phase separation occurs at the surface of the droplet. This crust formation significantly decreases the diffusion coefficients of volatile organic compounds relative to water. The newly developed skin allows water to diffuse (selective diffusion), but most importantly, it aids the retention of flavor chemicals.

The species and strain of the probiotic, inlet temperature, outlet temperature, speed of atomization, osmotic stress, dehydration stress, and wall materials affect the probiotic survival rate [28,29,30]. Different studies include the addition of wall materials for probiotic viability preservation. Hydrolyzed WPI+FOS (1.5:1) maintained the viability of *L. plantarum* at >10^8^ CFU/mL during 60 days of storage at 4 °C [31]. Similar behavior was described for skim milk, non-fat milk with xanthan gum, and WPI with dextran [32,33,34]. A viability of <10^9^ CFU/g after 24 weeks of storage at 25 °C and 11% RH for microcapsules of *L. rhamnosus* with WPI (20%), inulin (4%), and Persian gum (1%) was reported by Moayyedi et al [35].

The buffering capacity of wall materials provides a good shield for probiotics, hence protecting them against GI tract conditions [30,32]. Occasionally, the acid and bile tolerance of some bacteria enhances their survival.

### 2.2. Spray Cooling

Spray cooling is similar to spray drying. In this method, particles are formed by cooling and solidification of the droplets and not by evaporation of the solvent. In spray cooling, energy is removed from the droplets, causing the core material to solidify, whereas in spray drying, energy is applied to the droplets, causing the solvent to evaporate [36,37]. The bioactives are then dispersed in a liquid matrix material. Lipids or gel-like hydrocolloids can be encapsulating materials with this method. Solidification of the matrix is induced around the dispersed bioactives following cold air or liquid nitrogen spraying in a chamber. This leads to the formation of microcapsules. The feed, air cooler, cooling chamber, product collector, and fan form the main parts of the spray cooling process [4,36].

The low cost, continuous process, suitability for industrial scale make spray cooling an advantageous technique for food ingredient encapsulation. In addition, the non-use of organic solvents makes it environmentally friendly. However, its operation at relatively high temperatures may not enable it to be a suitable option for heat-sensitive ingredients such as omega-3 fatty acids, certain enzymes, and probiotics. Moreover, the relatively low encapsulation efficiency and the losses of bioactives during storage might cause limitations of this method [4,36].

*S. boulardii*, *L. acidophilus*, and *B. bifidum* have been encapsulated using the spray chilling or cooling technique [38]. The survivability of spray-chilled (*S. boulardii*, 97.89%; *L. acidophilus*, 83.57%; *B. bifidum*, 88.50%) and spray-dried (*S. boulardii*, 97.51%; *L. acidophilus*, 84.05%; *B. bifidum*, 90.10%) probiotics under simulated gastric conditions showed a great similarity. Probiotics have been encapsulated through the spray chilling technique in probiotic-enriched cream-filled cakes [39] and savory cereal bars [40].

### 2.3. Freeze Drying or Lyophilization

Lyophilization is a dehydration method suitable for the encapsulation of thermally sensitive substances. This technique consists of three stages [41]. In the first stage, ingredients are frozen quickly and thus ice crystals are created in order to limit the damage to the cellular structure. The freezing must be complete because any water that is not frozen cannot be removed by sublimation. The quality of the freeze-dried product is affected by the speed of freezing. In liquid foods, for example, slow freezing is recommended, where large ice crystals are formed which are joined together in various places. In solid foods, rapid freezing is recommended, where numerous and small-sized crystals are formed which do not join together.

In a secondary phase, the removal of water and therefore the dehydration takes place in two stages: (a) through sublimation (moisture reduction to ~15%) and (b) via evaporation (desorption) of the non-frozen water (moisture reduction to ~2%). For sublimation dehydration, the frozen ingredient is introduced into the lyophilization dryer. A vacuum chamber, a product heating system, and a water vapor condensation system comprise the dryer. Maintaining the pressure in the drying chamber below the water vapor pressure of the ice surface achieves continuous removal of water vapor from the product. A vacuum pump removes the water vapor. The pressure reduction immediately after the product enters the vacuum chamber should be carried out very quickly, along with low pressure in the vacuum chamber and the constant maintenance of the pressure during dehydration, otherwise, ice melting occurs and water forms.

In the third and final stage, heat is supplied to the product which is equal to the latent heat of sublimation of ice, i.e., it cannot be higher than the freezing point of the product and hence, the nutritional value of the product is not degraded. The required heat is supplied to the food via conduction, radiation, or a combination of the two methods, or even by the application of microwaves. In any case, the water vapor produced during the sublimation of the ice crystals is removed through the porous system created by the sublimation in the food, and then, the condenser of the dryer removes it before reaching the vacuum pump.

Finally, although the freeze-drying technique is successful for almost all food ingredients (with the exception of those that are difficult to dry), its cost seems to be five times the cost of spray drying. The encapsulation of water-soluble spirits, natural perfumes, and medicines is accomplished by this technique. Examples of applications are protein powders, lyophilized curcumin powder, etc., where their redispersion in water activates the activity of bioactives [41].

Freeze drying (FD) is the most common method for drying heat-sensitive ingredients in the food and pharmaceutical industries, maintaining a number of viable probiotics. The probiotic suspension freezes below its eutectic point, while the removal of the frozen water is achieved by the low pressure effect. The cell membrane is being damaged by the formation of ice crystals during the freeze-drying process. The cell membrane and cell proteins are stabilized by cryoprotectants such as lactose, trehalose, maltodextrin, sorbitol, sucrose, or milk proteins. Freeze-dried probiotics should remain active during storage. Storage conditions therefore play an important role in their viability, which generally appears high at temperatures < 4 °C and low at temperatures between 20 and 30 °C [24].

Freeze drying stabilizes probiotic bacteria after drying and storage, and more specifically, this technique stabilized *L. rhamnosus*, as reported by Moayyedi et al. [35], *L. acidophilus* [42], *Lactococcus lactis* [43], *L. plantarum*, and *L. acidophilus* [44]. Shu et al. [42] observed that *L. acidophilus* survival improved following the addition of sodium phosphate in the cryoprotective medium. A high viability is mentioned at conditions below 4 °C, whereas environmental temperatures (approximately 20 to 30 °C) commonly reduce probiotic viability. Moreover, encapsulation agents, probiotic strain, and storage conditions, such as water activity (*RH*) and temperature, might enhance probiotic viability, as reported by all of these studies mentioned above. Amounts between 7–~8 logs CFU/g of these probiotics have been discussed after exposure to simulated GI fluids.

### 2.4. Fluidized Bed Coating

Fluidized bed coating is used in the encapsulation of mainly sensitive components, in which spraying an encapsulating agent into a fluidized bed of powder produces coated particles. It is often combined with spray drying technology.

More specifically, a uniform layer is formed around the particles as the coating agent is deposited on the surface, thus enhancing the barrier properties and increasing the protection of sensitive components [45,46]. An air stream suspends the dust particles at a precise temperature and these are then sprayed with a coating material. Aqueous solutions of gums, starch, cellulose, and proteins could be coating materials, and these materials should have an acceptable viscosity, be thermally stable, and be able to form a film around the particle. The fluidized bed coating process involves three main steps: (a) the powder particles to be coated in the coating chamber need to be air stream fluidized, (b) the nozzle sprays the coating material onto the particles, and (c) the coating material sticks to the particles via evaporation of the coating material solvent with hot air. The most important parameters with an impact on agglomeration and particle formation affecting coating performance are humidity, spray pressure, coating feed rate, and temperature [47]. Fluidized bed coating is used in the nutritional supplement market to deliver encapsulated nutrients such as a variety of vitamins and minerals. In the meat industry, many acids have been encapsulated by this technique to improve color and flavor, as well as to reduce processing time. It is also used in bakery products to coat additives such as acetic acid, lactic acid, sorbic acid, potassium sorbate, calcium propionate, and salt [46].

High cost and direct exposure to high temperature, which can cause particle degradation and possible particle agglomeration, are some of the disadvantages of fluidized bed coating. Introducing more process parameters that could potentially affect the product properties makes the process much more complicated. However, the flexibility of this technique could easily be adopted for the mass production of biospheres, hence its use in the production of commercial dried yeast [24,25].

For probiotic encapsulation, a previous treatment of the cells is required to promote a solid particle which can be suspended and covered, characterizing the fluidized bed as a co-encapsulation technique [48,49,50]. Cellulose has been employed as an encapsulating material to encapsulate *Enterococcus faecium* IFANo.045 and *L. plantarum* IFANo.278 for the use of the fluidized bed technique, as reported by Strasser et al. [51]. They also emphasized that adding sucrose and trehalose to wall materials increased cell protection rates. Fluidized bed coating was also effective in producing alginate–chitosan microcapsules containing *L. plantarum* NCIMB 8826. This improved the storage cell survival of encapsulated probiotics compared to free cells, as reported by Albadran et al. [52].

### 2.5. Extrusion

Extrusion being a simple, low-cost encapsulation technology does not use harmful solvents, with efficient operation under mild conditions without advanced equipment [53,54]. It can be considered as an ideal technique for the encapsulation of thermosensitive bioactives, such as probiotics and ω-3 fatty acids. More specifically, this technique involves mixing bioactives into a hydrocolloid solution which is then extruded through a nozzle into a curing solution (e.g., CaCl_2_, AlCl_3_, or FeCl_2_). Thus, the initial mixture turns into a gel and pellets are created. The diameter of the nozzle, the distance between the nozzle, and the curing solution, as well as the concentration of the hydrocolloid solution, affect the size of the beads produced [7,26]. Various polysaccharides (most commonly sodium alginate) can be used for extrusion encapsulation, but the choice of these will determine the size, shape, and viability of the encapsulated material [55].

Extrusion is distinguished based on the operating method of the extruder, either hot or cold. Hot extrusion uses high pressures and temperatures. The rapid release of pressure as the food exits the dye causes the steam and gases to instantly expand through the product, forming a low-density food. Thus, a variety of different shapes such as spheres, tubes, sticks, etc., are obtained. In cold extrusion, the temperature of the food remains the same as that of the environment. In this case, the screw rotates at a low speed without developing friction and significant pressures, therefore the product does not undergo changes in its structure.

Hot and cold extrusion can be employed as a preservation method in order to reduce the water activity of the food. The main disadvantage of this technique used by the food industry is the formation of large particles and a slow production rate. This can be compensated for by modifying the technique with electrostatic air flow extrusion, rotating disk spraying, or using multiple nozzles simultaneously [26].

Haghshenas et al. [56] encapsulated *L. plantarum* 15HN using alginate, an alginate–psyllium blend, and alginate–fenugreek blend. To produce the beads, the solutions were extruded through a 21-gauge nozzle in a sterile calcium chloride solution. Etchepare et al. [57] used resistant starch, chitosan, and alginate to encapsulate *L. acidophilus* La-14 via extrusion in a calcium chloride solution using an aerograph coupled to an air compressor. Rodrigues et al. [54] used alginate combined with natural polysaccharides present in linseed and okra mucilages, botryosphaeran (exopolysaccharide produced by the endophytic fungus, *Botryosphaeria rhodina* MAMB-05), and fructo-oligosaccharides to encapsulate strains of *Lactobacillus casei* 01 and BGP 93 via extrusion. A protective effect on the cell viability of the encapsulated probiotic during 15 days of refrigerated storage was observed.

### 2.6. Ionic Gelation

An electrostatic interaction between opposite charges containing at least one polymer carries out encapsulation using this technique. The ionic gelation method is most often used to prepare alginate particles. Initially, an aqueous polymer solution containing low molecular mass ions (e.g., CaCl_2_, BaCl_2_, MgCl_2_, CuCl_2_, or ZnCl_2_) which react with oppositely charged electrolytes, resulting in an insoluble gel, starts ionic gelation. This mixture is added dropwise to a solution containing oppositely charged ions under vigorous and constant stirring. The formation of spherical particles due to the complexation between the oppositely charged ions is the initiation of ionic gelation. The spheres are then removed via filtration, poured with distilled water, and dried. It is a simple, cheap, and fast method with no requirement for special equipment, high temperatures, or organic solvents. However, it is considered to be disadvantageous due to the difficulty in producing uniform-sized particles [4,58].

The type and concentration of polymers used in ionic gelation determine the success of ionic gels [25]. Alginate particles (AMPs) were used for the encapsulation of *B. licheniformis* BCR 4–3 marine probiotics for improved storage stability and targeted delivery within shrimp intestines. The AMPs were able to effectively deliver probiotic bacteria that remained effective and stable [59]. The viability (from 3.28 log to 3.23 log CFU/mL) of the *L. acidophilus* (1643 PtCC) in a biliary salt condition for 120 min was sustained by Ebrahimnejad et al. [60] when they used chitosan and tripolyphosphate anions as wall materials.

The use of chitosan as a coating material on alginate beads containing the bacterium *Bifidobacterium longum* has been shown to improve its survival in gastric fluid and high-temperature conditions. However, the disadvantage of its use as an encapsulation material for probiotics is the inhibitory effect that it exhibits against microorganisms, including lactic acid bacteria [7].

Vaziri et al. [61] reported that the use of sodium alginate and alginate–chitosan blends crosslinked with Na-tripolyphosphate enhanced the encapsulation efficiency of *L. plantarum* from 97 to 100%. Different probiotics have been encapsulated via the ionic gelation process such as *L. casei*, *L. rhamnosus*, *L. plantarum*, *Pediococcus pentosaceus*, *L. fermentum*, and *L. acidophilus* [62,63,64,65,66,67]. Regarding the survival rate, it has been reported that it depends on the sensitivity of the probiotic strain. In a study by Rather et al. [68], *L. plantarum* had a survival rate higher than *L. casei.* Double crosslinking using Na-TPP improved the viability of *L. plantarum* (93%) compared with simple alginate crosslinking alone (85%), as shown by Vaziri et al. [63].

### 2.7. Complex Coacervation

Microencapsulation through coacervation is carried out in three stages via continuous stirring. In the first stage, the formation of three chemically immiscible parts takes place: the core medium, the assembly fluid, and the coating substance. In the second stage, the base substance is diffused into the coating solution. In the third stage, the outer layer is solidified via chemical or physical crosslinking reactions [24,69]. It is a low-cost technique which has a high loading capacity, has no release difficulties, does not require the use of organic solvents, and does not require the presence of extreme reaction conditions to harden the core shell. This process uses gum arabic (GA), chitosan, mesquite gum, pectin, alginate, xanthan gum, carrageenan, and carboxymethyl cellulose from polysaccharides, gelatin, whey protein, casein, albumin, and plant proteins such as pea or soy protein [24].

Yin et al. [10] encapsulated probiotics *Lactobacillus rhamnosus* GG in coacervates formed by whey protein isolate-high melting point fat shortening oil (SO) and gum arabic through complex coacervation and reported a higher survival rate of 54.96%. Complex coacervation leads to the formation of stable and strong network coacervates around the core materials, following the formation of the two-phase system based on the electrostatic attraction of two polyelectrolytes with opposite charges [70,71].

Different probiotics have been encapsulated via the complex coacervation process such as *L. plantarum*, *casei*, *paracasei*, *B. lactis*, *L. acidophilus*, and *L. reuteri* [72,73,74,75,76,77]. Regarding viability, Bosnea et al. [74] found that the encapsulation of probiotics in WPI:GA coacervates grew slightly faster than free bacteria under simulated gastric conditions, whereas Mao et al. [75] reported that the preservation of *B. longum* under simulated GI conditions was accomplished via soy protein microcapsules isolated from *i*-carrageenan (ratio of 10:1).

### 2.8. Electrospray and Electrospinning as Part of Nanoencapsulation

Nanoencapsulation is a strategy ultimately leading to the entrapment of a core component into a protective nanocarrier. Several strategies can be employed to carry out the process. Antisolvent precipitation, electrospinning, electrospraying, the emulsion–evaporation method, high-pressure microfluidization, ionic gelation, lyophilization, nanoemulsification, nano spray drying, and ultrasonication-assisted emulsification are some of the technologies used in the production of nano-encapsulated materials [78,79,80,81]. Many factors such as the core component and wall material properties, as well as the interaction between them and the intended application, might affect the selection of the method.

Nanoencapsulation in the context of sustainable food production is described in Table 1, for example, the incorporation of hydrophobic compounds in foods due to reduced miscibility among food components.

The use of gelatin nanofibers for the improvement in the solubilization of lycopene was explored by Horuz and Belibağlı [86] who found a significant increase in the solubility in aqueous solution by encapsulating it with gelatin nanofibers. 

A similar approach was explored in a study with cheese crackers [87]. The nanoparticles of sodium chloride were produced by nano spray drying and applied to the surface of samples (1, 1.5, and 2% of sodium chloride). Higher scores for saltness than the control (2% of sodium chloride) were received in fresh samples. Higher scores in saltness were reported in samples produced with nanoparticles compared to the control after 4 months of storage. A similar experiment was carried out with potato chips by Vinitha et al. [88], where the sodium chloride nanoparticles were generated via an electrohydrodynamic atomized drying method. The perception of saltness in relation to the control chips with commercial size sodium chloride was increased by the use of nanoparticles, and sodium chloride was reduced by 65% [89].

During the nanoencapsulation of probiotics, a protective nanoshell is formed that aims to surround them. Nanocapsules, nanoemulsions, nanoliposomes, and nanoparticles are the most common formations. Nanocellulose, starch nanoparticles (SNPS), titanium dioxide, silicon dioxide, and zinc oxide nanoparticles are also the most commonly used nanomaterials. The nanoscale coating limits the loss of integrity of the probiotics until they reach the gut while still managing to transport and reach their target under adverse conditions, as reported by authors in Table 1.

Electrospraying and electrospinning are electrohydrodynamic (EHD) techniques, characterized as simple flexible and mild techniques managing to attract interest for encapsulating and immobilizing probiotics. From these emerging techniques, nano/microscale fibers and spheres are produced [90]. A needle using a syringe pump carries out the extrusion of the polymer solution. The formation of a Taylor cone at the tip of the needle takes place following the emergence of the droplet. Explosion of the jets out of the cone then occurs, followed by collection of the broken droplets by the collector [91].

Xu et al. [92] reported an increased survival rate of 89.26% after electrospinning and an increased survival rate of 84.63% after 21 days of storage of *Lactobacillus rhamnosus* 1.0320 when they used poly(vinyl alcohol) (PVA)/pectin (PEC) as the wall material. Survivability of 90.07% and 91.96% in simulated gastric and intestinal fluids of *Lactobacillus rhamnosus* 1.0320 was maintained by Xu et al. [93] when they used PVA/PEC. Ma et al. [94] managed to enhance the stability of cells of *L. plantarum* KLDS 1.0328 and GA when they used PVA as the wall material. 

### 2.9. Emulsification 

Emulsification is commonly used to encapsulate lipid bioactives. The dispersion of the encapsulated material (discontinuous phase) in an organic phase (continuous phase) takes place here via the formation of an oil-in-water emulsion. The emulsion is prepared by homogenizing the mixture with the help of surfactants [17,58]. Often, it is used as a preparatory step for other techniques, e.g., ionic gelation, spray drying, etc.

### 2.10. Encapsulation in Yeasts

The application of yeast cells as encapsulation agents has become evident in recent years. The most obvious benefit of this application is that the cell itself takes the shape of an oval capsule that can be used as it is. The thick (100–200 nm) and β-glucan-rich cell wall as well as the plasma membrane provides the robust structure, thus holding both hydrophilic and hydrophobic compounds within its bulk. The diffusion of compounds according to their molecular weight and hydrophobicity are allowed due to the porosity of the cell wall, and this provides good mechanical strength. In addition, yeasts are easier to cultivate on an industrial scale [37]. Incubation of the cells with the material to be encapsulated forms a simple impregnation process, and this has been described by Dimopoulos et al. [95], showing benefits of the encapsulation of compounds inside the cell. They encapsulated the essential oil of oregano in cells of *Saccharomyces cerevisiae* in order to cover its strong smell but also to protect its bioactives from external processing parameters.

A wide range of applications, such as food, medicine, or cosmetics, use yeast cell capsules. Yeast cells are not empty in their natural state. Cytosol removal can be carried out via extraction, hence the flavor of the yeast itself is reduced and more space is provided for the encapsulation of valuable substances. The cell wall is the way for substances to be encapsulated in yeast cells since they must pass through that to enter the cells [96]. This can be achieved in the following ways [95]:

- Autolysis: The permeability of yeast cells can be increased by this method or cells can be broken with the aim of obtaining intracellular products. When the cell’s own enzymes proceed to hydrolyze its macromolecules, this is called yeast autolysis. The cell wall structure is affected only in terms of permeability and retains its shape after the procedure is completed, as evidenced by electron microscopy. The main damage observed is located in the plasma membrane. In the food industry, autolysis takes place under conditions of temperatures of approx. 50 °C and pH = 5.5.
- Pulsed electric fields: The membrane of the yeast cells is exposed to an external high-voltage electric field, thus resulting in the formation of pores on the surface of the membrane, and this is called pulsed electric field treatment. Moderate electric field strengths of yeast cells do not cause cell disruption but lead to increased cell permeability at both the cell membrane and cell wall level. However, damage to the cell such as gaps in the cell wall and shrinkage of the cytoplasm is caused at very high electric field strengths (40 kV/cm). This technique has been studied in yeast cells with the aim of increasing the extraction of yeast extract, polysaccharides, proteins, and enzymes.
- High-pressure homogenization: It is a method to break up cells in large-scale processing. Intracellular products such as proteins from *E. coli* and *S. cerevisiae* can be obtained by this method. Being a non-thermal method, the pressure of the cell suspension is increased up to a pressure of 1000 bar, hence a specially designed valve system of the homogenizer releases the suspension. Cells receive a wide range of forces that can even cause their complete lysis and, by extension, an increase in cell permeability.
- Plasmolysis: plasmolysis involves cell incubation in the presence of NaCl or ethyl acetate, causing the cell membrane to rupture due to osmotic stress.

The conventional process for encapsulation can be divided into three main steps: mixing of the yeast and bioactives in water at a certain period allowing the substance to migrate into the cells, removal of any unencapsulated bioactives in order to wash the cells, and spraying or lyophilization so that the drying of the cells containing bioactives is accomplished, hence acquiring the binding of the substances inside the cells.

The cell wall pores close again and the substances remain trapped inside them when the yeast cells are dried [96].

The immobilization of yeast cells is currently used in industrial winemaking (i.e., sparkling wine and fino sherry wine) and brewing. The production of sparkling wine has been accomplished by yeast cells encapsulated in alginate beads, as reported by [97,98,99].

Until now, the production of fermentation beverages such as white wine, sparkling wine, natural sweet wine, beer, red raspberry wine, and bioethanol from molasses or starch has been achieved by the use of biocapsules [100,101,102,103,104,105,106,107,108,109].

However, a new methodology to assemble biocapsules for the infusion and immobilization of yeast cells into filamentous fungal pellets, which serve as a porous natural material, has been reported to cover the restriction of the microorganisms’ growth [110]. Yeast cells can be forced towards the core of the fungal pellets followed by culture in a YPD medium to promote their growth from the interior via vacuum application.

This technique involves four steps: (i) separation of yeast cells and culture filamentous fungal spores to promote biomass production and avoid microorganism competition, (ii) increase yeast cell population in the pellet core by mixing the resulting pellets and yeasts suspension, and yeast cell infusion into the pellets through a vacuum, (iii) YPD liquid medium culturing to promote further attachment of the yeast and pellet, and (iv) removal of non-attached cells in the pellet surface by washing. The biocapsules formed have been named “microbial biocapsules’’.

### 2.11. Liposomes

**Figure 1:**
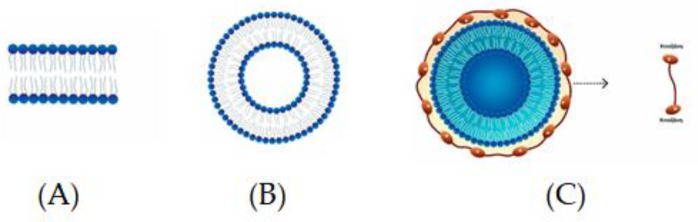
(**A**) Lipid bilayer and (**B**) liposome [111]; (**C**) liposome coated with chitosan.

Spherical structures composed of bilayers of phospholipids, which are distinguished by a polar head or hydrophilic unit and two hydrocarbon hydrophobic tails, are called liposomes (Figure 1A,B) [111]. The encapsulation of food components of different polarity, so that a natural barrier is formed and bioactives are protected from external conditions, can be carried out via the presence of liposomes due to their amphiphilic nature and their simultaneous occurrence [111]. Some of the distinct advantages of liposomes in encapsulation are as follows: (a) better physical stability, (b) controllable particle size, (c) ease of preparation and the possibility of industrial-scale production, (d) the encapsulation of both lipophilic and hydrophilic components, (e) the controlled and sustained release of the ingredients, (f) the non-use of organic solvents, and (g) the cost-effectiveness [112].

The thermodynamic instability and the disruption of their structure during food processing, causing disintegration and release of entrapped compounds, are some of the limitations of liposomes. Hence, the evaluation of the behavior of the particles over time under different conditions such as pH and temperature is being studied by stability studies. Controlled release of the encapsulated ingredients and in vitro studies are conducted to test the release of these ingredients in GIT conditions in stability studies. Many authors propose the application of biopolymers as coating materials such as chitosan to be widely used for coating liposomes to modify the surface of liposomes in order to make them more protected, stable, and consequently applicable. Examples of other biopolymers that are also frequently used are starch, alginate, whey protein, and gelatin [111]. The coating of liposomes with biopolymers, such as polysaccharides and proteins, is well known as surface nanoengineering (modification of the surface). Protective films such as food-grade natural biodegradable polymers are used as a novel approach to nanoencapsulation in the food manufacturing sector [113]. In this direction, gelatin (GE)–chitosan (CH) polyelectrolyte-coated nanoliposomes were developed and characterized by Adeel et al. [114] to prolong the viability of probiotics (*L. acidophilus*). Hence, these nanoliposomes could be used as an effective carrier for the delivery of probiotics.

#### Chitosomes

A novel way to improve the encapsulation efficiency of liposomes is to modify the surface of the liposomal membrane through the formation of bioadhesive and polymeric layers. Chitosan is a linear natural polysaccharide widely applied in biomedical and biotechnological fields due to its biocompatibility, bioadhesiveness, biodegradability, and non-toxicity. Hence, the development of phospholipid–chitosan composite vesicles (chitosomes) as novel structures for the encapsulation of drugs and nutrients is of interest [115] (Figure 1C).

Chitosan can be obtained via partial deacetylation of chitin. In an aqueous solution with a pH < 6.5, the amines of chitosan are protonated. They can be applied as a coating material due to the electrostatic attraction between the protonated amino groups of chitosan and the negatively charged groups present on the surface of liposomes. Chitosomes form thick layers around liposomes with the immediate result being an increase in particle size. For chitosomes preparation, it is usually recommended to produce liposomes normally and then promote the electrostatic deposition of cationic chitosan on the liposome surface via the addition of a chitosan solution under stirring [111]. Alginate–chitosan has been used for the encapsulation of *Lactobacillus rhamnosus* ASCC 290 and *L. casei* ATCC 334, as reported by Farias et al. [116]. This provided an encapsulation efficiency of >76%, and both bacteria were protected under simulated gastrointestinal conditions.

### 2.12. Inclusion Complexation

This method is performed with cyclodextrins (CDs) as encapsulating materials. CDs are cyclic oligosaccharides derived from starch via enzymatic cleavage. α-, β-, and γ-CDs have 6, 7, and 8 glucose units, respectively, covalently attached. Their characteristic is the hydrophobic inner cavity and the hydrophilic outer surface [37]. Structural differences make the physicochemical properties of α-, β-, and γ-CDs differ slightly. A relatively small molecular cavity, so it can only accept small molecules, makes α-CD research limited. The larger molecular cavity but high production cost and its non-suitability for large quantities are characteristics of γ-CD. β-CD is simple and economical to produce on a large scale, with a reasonable cavity size, and is applied to commercialized foods.

The only encapsulation method that occurs at the molecular level is CD encapsulation. CD can neutralize characteristic odors, e.g., of garlic and onion oils, complexing their volatile components. The formation of complexes with CD allows the controlled release of bioactives and may prove useful for shelf-life optimization and the bioavailability of encapsulated compounds. It has also been reported that CD enhances the stability of fat-soluble vitamins A, E, and K [4]. Finally, there are many encapsulation methods using cyclodextrins such as spray drying and lyophilization. 

### 2.13. Hydrogels

Protein–polysaccharide composites have constructed hydrogel systems, and this has attracted researchers’ attention due to their biodegradability, biocompatibility, and non-toxic properties. Soy protein isolate–citrus pectin composite hydrogels induced by transglutaminase (TGase) and ultrasonic treatment for 20 min protected the survival of *Lactiplantibacillus plantarum* (new name of *L. Plantarum*) from gastrointestinal digestion and UV irradiation, as reported by Liu et al. [117]. Moreover, Zhang et al. [14] constructed double-saccharide composite hydrogel beads for the encapsulation of *Companilactobacillus crustorum* MN047 by applying different combination ratios of low methoxyl pectin (LMP) and sodium alginate (SAG) and the concentrations of calcium chloride (CaCl_2_).

In Table 2, the advantages and disadvantages of each encapsulation technique are described. 

## 3. Co-Encapsulation of Probiotics

Probiotics are part of functional foods and include lactic acid bacteria, *lactobacilli*, and the *Lactobacillus* and *Bifidobacterium* family but also non-bacterial organisms (non-pathogenic yeasts such as *S. boulardii*. *Lactobacillus* and *Bifidobacteria* which are Gram-positive, anaerobic bacteria that produce lactic acid, and other antimicrobial compounds, e.g., hydrogen peroxide and peptides with bacteriostatic activity. Clearly, there are other genera that exhibit probiotic properties and have been commercialized such as *Streptococcus*, *Bacillus*, *Enterococcus*, *Saccharomyces*, *Leuconostoc*, *Pediococcus*, and *Lactococus* [118,119,120].

Probiotics serve several functions in the human body, among which is maintaining a balance between pathogenic and non-pathogenic bacteria that are necessary for the normal functioning of the organism. Their main advantage is that they can effectively limit the growth of harmful bacterial strains such as *Escherichia coli*, *Clostridium perfringens*, *Shigella sonnei*, *Salmonella enteritidis*, *Campylobacter jejuni*, *Staphylococcus*, and *Yersinia* [120]. According to Telessy [121], lactose intolerance, ulcerative colitis, Crohn’s disease, *Helicobacter pylori* infection, metabolic diseases, respiratory system infections, allergies, and mental/neurological illnesses constitute the main conditions/diseases where the use and consumption of probiotics are indicated. Based on the World Gastroenterology Organization, the required dose of probiotic microorganisms depends on the strain and the product. Many commercially available products contain between 1 and 10 billion colony-forming units (CFU) per dose, whereas some formulas have shown efficacy at lower levels, while others require higher amounts. At least 10^6^–10^7^ CFU/g of probiotic bacteria is the dose for a probiotic product in order to show a therapeutic effect based on a minimum consumption of 100 g or 100 mL of food [17].

### 3.1. Co-Encapsulation of Probiotics with Bioactive Substances

Encapsulation of bioactives and probiotic bacteria in a single product can enhance the bioactivity of the individual ingredients but also provide synergistic health benefits. This process is governed by the advantages of lower cost and convenience over the microencapsulation of individual ingredients, and precisely for these reasons, it has been widely used in the pharmaceutical industry. The co-encapsulation technique has also enhanced the long-term storage capability of food products while maintaining the stability of probiotics and bioactives. However, more research is needed regarding the mechanism of release of these ingredients in simulated animal system conditions, as well as the creation of more cost-effective functional food products [19].

In addition, the selection of bacteria for use as probiotics is based on certain parameters. First, probiotics must be safe for consumption, which depends on the species, strains, isolation conditions, and antibiotic resistance. Second, probiotics should be able to be produced economically in high quantities and remain stable in food and the GIT. And finally, a necessary criterion is that they can colonize the intestine and promote the creation of a healthy microbiome. Typically, these beneficial bacteria belong to the genera *Lactobacillus* and *Bifidobacterium* which include a variety of species that have been generally recognized as safe [122,123]. 

**Figure 2:**
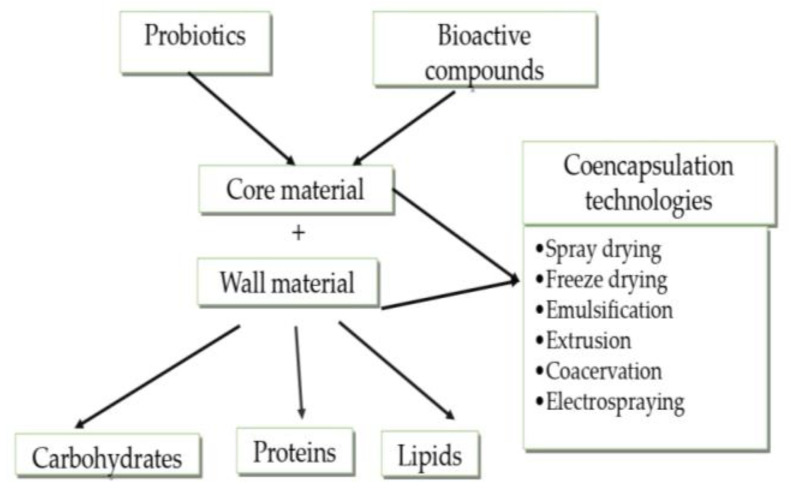
Co-encapsulation technologies for combination of probiotics and bioactive substances (adapted from [7,19]).

The main techniques used for co-encapsulation are spray drying, freeze drying, extrusion, coacervation, electrospraying, and emulsification (Figure 2) [7,18]. Often, to achieve more efficient results, these techniques are combined with each other or with others. For example, emulsification has been combined several times with ionic gelation.

#### 3.1.1. Co-Encapsulation of Probiotics with Prebiotics

One of the most important reasons that probiotic encapsulation has a low success rate is their inability to survive within the encapsulation material. The co-encapsulation of prebiotics (such as inulin, polydextrose, fructo-oligosaccharides, and galacto-oligosaccharides) together with probiotics promises greater success in terms of the viability and stability of these live microorganisms. Some researchers have stated that prebiotics may improve the ability of probiotics to survive during their passage through the GIT and, at the same time, enhance their biological effects in it [17,124]. The main biocompatible materials for the purpose of co-encapsulating synbiotics are alginate, chitosan, pectin, gelatin, starch, gum arabic, whey protein, and lipid carriers, as well as various mixtures of these materials. It should be noted that the beneficial effects of probiotics and prebiotics depend on their appropriate combination, which requires the examination of the bacterial strain and its antimicrobial activity. The synbiotics created can help balance the gut microbiota and fight multi-resistant organisms [18].

Co-encapsulation of probiotics with prebiotics, such as inulin, resistant starch [125], fructo-oligosaccharide (FOS) [126], and arabinoxylan [127], has been shown as a promising alternative to facilitate the oral delivery and release of viable probiotics to their targeted site (i.e., the host colon).

Co-encapsulation of probiotics with prebiotics such as inulin and polydextrose preserves their viability during the GI transit. Moreover, bacteria are prevented from detrimental conditions such as low pH and oxidative stress during processing and storage via the addition of antioxidants such as tocopherol and ascorbic acid in microcapsules [128,129].

#### 3.1.2. Co-Encapsulation of Multiple Probiotics

There is evidence that mixed forms of probiotic cultures are more effective than single strains. For example, the mixture of *Streptococcus thermophilus*, *Lactobacillus acidophilus,* and *Bifidobacterium bifidum* is more effective than the individual strains in reducing cholesterol and triglyceride levels in a study conducted in vivo [122]. It has also been found that peptides and amino acids produced by *Lactobacillus bulgaricus* can enhance the growth of *Streptococcus thermophilus*. In one study, the bacteria *L. plantarum* and *Bifidobacterium lactis* were separately co-encapsulated with either inulin or resistant starch in calcium alginate/chitosan microcapsules. Promising results were obtained to enhance the survival of probiotics during the period of their storage and passage through the GIT [125]. In a similar study, *L. bulgaricus* and *Lactobacillus paracasei* were co-encapsulated in alginate-coated whey protein pellets. The specific biopolymer pellets proved to provide better resistance of the two strains to heat treatment, as well as to simulated GIT conditions. In general, between two different bacterial strains, there can be either competition or co-operation, so it is important to choose appropriate combinations of probiotic bacteria [123].

Co-encapsulation of cryoprotectants and porous starch within calcium alginate capsules (CAC) with *Lactiplantibacillus plantarum* grx16 showed that the viability of probiotics encapsulated could be improved to 82.68%, as reported by Zhou et al. [130]. 

#### 3.1.3. Co-Encapsulation of Probiotics and Omega-3 Fatty Acids

Sultana et al. [131] co-encapsulated *L. casei* and tocotrienol-enriched flaxseed oil in calcium alginate–carboxymethyl cellulose hydrogel beads (CA-CMC) for the delivery of a functional orange juice. Different techniques have been reported by Sultana et al. [120]. In this context, the co-encapsulation of *L. plantarum* and DHA-rich oil in alginate–pectin–gelatin microcapsules via the extrusion-dripping technique has been mentioned by Vaziri et al. [132], and *L. plantarum* and flaxseed oil in sodium alginate–pectin microcapsules via the same technique has been stated by Lopez-Fernandez et al. [133].

## 4. Controlled Release Mechanisms of Probiotics

Although the main goal of encapsulation is to protect the internal phase from adverse conditions, a very important parameter is also the release of bioactives, at the right time and at the right place. The interaction of bioactives with the wall material, along with the size of the microparticles, their volatility, the viscosity of the encapsulation vehicle, and the ratio between the core and wall material directly affects the release mechanism of bioactives [134]. The terms “bioaccessibility” and “bioavailability” are mentioned in this release. “Bioaccessibility” is defined as a bioactive fraction released from the original site (food or encapsulation material) in the gastrointestinal tract (GIT) [135]. Accordingly, “bioavailability” is a constituent or ingested ingredient fraction that enters the circulatory system and is accessible for biological activities [136]. Therefore, the release of bioactives from their initial state in the delivery system is determined by these two properties of the coating materials. This release could be immediate or modified. Regarding immediate release, it could take place shortly after use. However, with regard to modified release, it can occur either for a prolonged period of time after use or at a specific organ within the body [137]. The survival of probiotics during industrial production, storage, and consumption can be improved by the encapsulation of microorganisms with probiotics [7]. Only under these conditions can they manifest their beneficial properties for the host [11]. Adverse environmental conditions can potentially cause cell damage and loss [1]. For example, as probiotics are transported and stored in inappropriate conditions with high temperature and humidity values, their viability is affected. Moreover, as probiotic bacteria are characterized as microaerophilic or anaerobic, high oxygen levels are also expected to reduce their viability [12].

**Figure 3:**
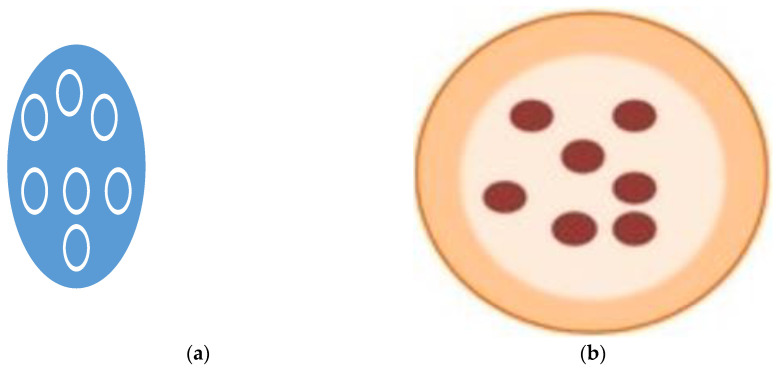
Release mechanisms for encapsulated probiotics leading to diffusion, swelling (**a**), fragmentation, dissolution, or erosion and (**b**) represents a capsule (adapted from [7]).

One type of the modified release systems is controlled release (CR) systems. This means that a specific rate and time regardless of local conditions are employed for the release of bioactives [138]. Through the consumption of probiotics, there is a risk of reduced usefulness in the human body, as the extremely low pH of gastric fluids of the stomach (usually ranging between 1 and 3) jeopardizes their viability and negatively affects a number of probiotics. Therefore, before they are consumed and transported to the human intestine, it is necessary to develop a system that will allow their gradual distribution [139]. The controlled release method for encapsulated compounds has the advantage of minimizing ingredient losses during processing. Diffusion, swelling, fragmentation, degradation, dissolution, and surface and bulk erosion are some common release mechanisms of bioactives (Figure 3) [37,138,140,141]. Probiotics for food can be delivered by particles, emulsions, beads, electrospun hybrid nanofibers, microcapsules, hydrogels, and bigels [139]. These mechanisms also release flavors from the encapsulation matrix [142]. The release of active material may be a combination of the above-mentioned release mechanisms [143].

Bioactive characteristics (initial amount, size, solubility, diffusivity, partition coefficient, and concentration gradient), carrier (matrix) properties, or encapsulation systems (original size, shape and structure, porosity, pore formation, and closure and (non)flocculated state of emulsion droplets) are the main parameters affecting controlled release [138,140], along with other factors such as the surrounding environment and GIT conditions (mouth, bolus going into the stomach, chime going to the small intestine, and indigestible ingredients going to the colon).

Fragmentation: The encapsulation carrier breaks due to different environmental conditions such as changes in pH, pressure, enzymatic activities, shearing changes, etc., and the encapsulated components are released. The fracture characteristics of the encapsulation system, including the imposed stress at the breakpoint, control the release rate. Other factors such as the shape and size of the fragments formed can also affect the release rate. Diffusion, dissolution, or erosion can be used to release bioactives from fragments. Faster release rates are predicted due to the generation of smaller fragments and the consequent increase in contact surface [138,140].

Swelling: Here, the release is being carried out via absorption of the solvent and therefore swelling of the encapsulation system. Both the rate of swelling and the time required for the food ingredient to diffuse through the swollen system govern the release rate [140]. Selecting the appropriate polymeric matrix, as well as controlling the surrounding conditions including temperature and pH, can affect the release mechanism of swelling [138,140].

Diffusion: The solubility and permeability of bioactives govern the process of diffusion. It is the dominant mechanism in controlled release systems and therefore the most important. It is the random movement of bioactives from the inside to the outside of the carriers, i.e., the dispersion of the molecules from the area of higher concentration (capsule) to the area of lower concentration. Diffusion is lower in solids and faster in gases and is affected by the properties of the polymer. Finally, diffusion might affect the stability of the encapsulation system, but this might change due to swelling, shrinkage, erosion, or fragmentation [134,138,140].

Erosion: Erosion is the release phenomenon without transport events. This can be differentiated into surface erosion, in which only the outer part of the carrier is degraded, and bulk erosion, in which the whole degradation is due to the whole carrier [62]. Erosion starts only when the degradation products of the polymer diffuse into the release environment and is shown as mass loss of the polymer [144,145].

Degradation: In this case, the polymer (homogeneous or heterogeneous) is degraded and all compounds are released from the carrier. More specifically, in a heterogeneous system, a reduction in the release rates of the substances is caused by the weakening of the surface of the carrier over time, since a higher proportion of core compounds is enclosed in the interior coatings compared to the exterior ones. In the homogeneous system, the degradation of biodegradable compounds is carried out gradually in the initial stage. However, in the later stages, acceleration of the degradation rate occurs due to autocatalysis events, and most of it is eroded in a relatively short period of time. Usually, heterogeneous erosion occurs in hydrophobic polymers. In hydrophilic polymers, homogeneous erosion usually takes place [138].

Multiple layer coating of biopolymers is a promising strategy for the CR of probiotics [146,147,148,149,150].

Basu et al. [151] focused on direct entrapment, internal and external microencapsulation of *L. casei* in a biopolymer (alginate) matrix, and their performance in lactose-rich media. They concluded that by using the burst-release mechanism, the model could predict the release time of *L. casei*—868 min, 587 min, and 574 min from alginate beads. Burst-release of the encapsulated cells in a synbiotic environment has also been discussed by Banerjee et al. [152]. 

Probiotic delivery systems with different trigger mechanisms have been constructed to successfully introduce numerous high-viability probiotics to the intestine, as discussed by Luo et al. [153]. They reported the oral delivery of probiotics based on a pH-sensitive trigger mechanism, based on mucosal adhesion, enzyme degradation, spore or biofilm formation, or a redox-responsive trigger mechanism.

Misra et al. [154] discussed novel microencapsulation techniques of probiotic bacteria and characterized microcapsules along with their mechanism of release and stability. They have discussed the application of microencapsulated probiotics in different foods such as fermented foods (kefir, fermented meats, and beverages), cereal-based products, bakery products, frozen desserts, dairy products, and extruded products (pasta, noodles, and others). For example, Li et al. [155] observed that the addition of microencapsulated probiotics *Lactobacillus* strains with galacto-oligosaccharides and lactitol via extrusion showed improved viability of 9.95 log CFU/g but an adverse effect on textural properties and syneresis of yogurt samples during storage compared to the control sample. Fermented ice cream affects the metabolic activity of probiotic bacteria; however, the loss in viability of strains in frozen dairy desserts are caused by frozen injury, oxygen toxicity, and higher osmotic pressure [156].

Finally, the addition of microencapsulated bacterium *S. thermophiles* into encapsulants (pectin, carboxymethylcellulose, cellobiose, and gum arabic) via emulsification showed good viability in dark (6.90 log CFU/g) and milk (7.12 log CFU/g) chocolate stored at 4 °C up to 180 days, with no effect on the sensory attributes and moisture content of the chocolate, as reported by Ozturk et al. [157].

## 5. Conclusions and Recommendations for Further Work

The ever-increasing demand from all stakeholders involved in food production for green approaches makes the encapsulation of probiotics an extremely promising approach. In addition, consumers are looking for probiotics-based foods that, on one hand, have beneficial properties for their health and on the other hand, have been produced without the use of intensive intervention processes that may leave residual residues in the food and degrade its nutritional value. The encapsulation techniques have proved to contribute to the increase in the effectiveness of many probiotics and the protection of their viability.

Probiotics linked to healthy gut maintenance and a strong immune system signify rewarding opportunities for the food industry. The consumption of probiotics in enriched or functional foods is an easy way to promote and maintain health and wellness while avoiding pills or medication. Probiotic foods are also sources of other nutritional compounds, such as antioxidants, fiber, unsaturated fatty acids, minerals, or vitamins and offer synergistic effects to health.

The development of new fermented and non-fermented foods for probiotic supplementation is part of food industry innovation in order to avoid pill fatigue and encourage the consumption of probiotics as part of a regular diet. Probiotic encapsulation has been proposed to overcome problems such as the inclusion of free probiotics into foods, which could alter the physicochemical characteristics, sensory properties, shelf life, probiotic viability, and functionality of the food.

The formulation and encapsulation process determines the storage viability, survival under gastrointestinal conditions, and functionality of microencapsulated probiotics. Future research should focus on new materials and processes to protect probiotics from high processing temperatures, such as pasteurization, sterilization, heating, and baking.

More studies are necessary to confirm the thermal protection achieved, for example, by the addition of lipids, the use of trehalose, or the hardening of the capsule in complex coacervation.

Although some of the studies are promising, the next step to evaluate the usefulness of probiotic encapsulation is clinical trials, despite dynamic gastrointestinal models having been developed to provide valuable information on the delivery and impact of food products on the human intestinal microbiota. The incorporation of probiotics into dairy/non-dairy and bakery products is a challenging issue that the food industry is facing nowadays, hence many issues need to be resolved before we see them in the market. Obstacles remain on the path to commercialization. Despite promising laboratory-scale results, difficulties emerge when scaling up the technology for industrial applications.

## Figures and Tables

**Table 1:** Nanoencapsulation of probiotics and their applications in sustainable food production.

Encapsulation Process	Probiotics	Wall Material(s)	Effect	Reference
Νanocoating	*Lactobacillus acidophilus*	Artificial nanoshells	Enhanced viability of nanocoating *L. acidophilus* in simulated gastric fluid (SGF) by 49% compared with free probiotics.	[82]
Electrospinning	Probiotic strains of LAB and *Bifidobacteria*	Corn starch (CS) and sodium alginate (SA) nanofiber	After a 20-day storage of yogurt, the count of nanoencapsulated LAB and bifidobacteria declined by 0.19 (97.9% survival) and 0.28 (96.9% survival) log CFU, respectively.	[83]
Electrospinning	Probiotic strains	Starch/sodium alginate	Enhanced viability rate of lactobacilli and bifidobacteria strains in the acidic environment and simulated gastrointestinal conditions.	[84]
Layer-By-Layer Coating	*Lactobacillus plantarum* 90 (LP90)	Whey protein isolate fibrils (WPIFs), sodium alginate (ALG), carboxymethyl cellulose (CMC), and xanthan gum (XG)	Enhanced survival of *Lactobacillus plantarum* 90 (LP90).	[85]

**Table 2:** Advantages and disadvantages of encapsulation techniques of probiotics.

Advantages	Disadvantages	References
Spray drying		
Short drying time; Low cost; Flexibility; High productivity process; Large-scale production; High speed.	Decreased number of viable probiotic organisms; Wall material must be soluble in water; Particles with irregular geometry and porous surface; Process losses of heat-sensitive substances have not been fully addressed.	[2,24,25,28,36,59,88]
Spray cooling		
Low-cost, fast process; Dense, spherical, and smooth particle surface; It can be applied on an industrial scale.	Low performance; Not suitable for heat-sensitive bioactives; Possibility of loss of bioactives.	[4,36]
Freeze drying or lyophilization		
Mild conditions; Suitable for heat-sensitive compounds; Almost unchanged nutritional value; Rapid comprehensive dehydration.	Decreased number of viable probiotic organisms; High cost of installation and operation of the equipment; Unsuccessful on foods that are difficult to dehydrate; Risk of cell damage.	[41,88]
Fluidized bed coating		
Suitable for thermally sensitive components; Mass production of biospheres.	Complicated process; Direct exposure to high temperature can cause molecules to degrade; High cost.	[24,25,45]
Extrusion		
Simple, low-cost technology; Mild conditions; Ideal for thermosensitive bioactives.	Reduced viability after the extrusion process; Formation of large particles; Slow production rate; Not applicable on an industrial scale; The use of hydrocolloid solution is necessary.	[25,26,36]
Ionic gelation		
Simple and economical method; No special equipment or organic solvents are required; Mild conditions.	It is not easy to produce particles of uniform size;	[4,7,58,60]
Emulsification		
Easy to scale up; Simple and flexible technique; High sustainability of bioactives, e.g., probiotics.	Not suitable for mass production; Non-uniform particle shape and size.	[16,25,59,88]
Encapsulation in yeasts		
Simple and low-cost process; High encapsulation efficiency; Good mechanical strength and easy cultivation on an industrial scale; No additional materials are used.	Mainly used for the encapsulation of lipophilic molecules.	[37]
Liposomes		
Encapsulation of hydrophilic and hydrophobic molecules; Production of uniform pellets on an industrial scale; No use of organic solvents.	Thermodynamically unstable method; The structure of the liposomes can be disrupted and the encapsulated substances can be released.	[111,112]
Inclusion complexation		
Economical to produce it on a large scale (β-cyclodextrin); Also suitable for volatile compounds; It allows the controlled release of bioactives.	High doses of cyclodextrin may be harmful.	[4,37]
